# Racial and Ethnic Disparities in Breastfeeding Initiation ─ United States, 2019

**DOI:** 10.15585/mmwr.mm7021a1

**Published:** 2021-05-28

**Authors:** Katelyn V. Chiang, Ruowei Li, Erica H. Anstey, Cria G. Perrine

**Affiliations:** ^1^Division of Nutrition, Physical Activity, and Obesity, National Center for Chronic Disease Prevention and Health Promotion, CDC; ^2^Oak Ridge Institute for Science and Education, Oak Ridge, Tennessee.

Breastfeeding is the optimal source of nutrition for most infants (*1*). Although breastfeeding rates in the United States have increased during the past decade, racial/ethnic disparities persist (*2*). Breastfeeding surveillance typically focuses on disparities at the national level, because small sample sizes limit examination of disparities at the state or territorial level. However, birth certificate data allow for assessment of breastfeeding initiation among nearly all newborn infants in the United States both nationally and at the state and territorial levels. To describe breastfeeding initiation by maternal race/ethnicity,* CDC analyzed 2019 National Vital Statistics System (NVSS) birth certificate data for 3,129,646 births from 48 of the 50 states (all except California and Michigan^†^), the District of Columbia (DC), and three U.S. territories (Guam, Northern Mariana Islands, and Puerto Rico). The prevalence of breastfeeding initiation was 84.1% overall and varied by maternal race/ethnicity, ranging from 90.3% among infants of Asian mothers to 73.6% among infants of Black mothers, a difference of 16.7 percentage points. Across states, the magnitude of disparity between the highest and lowest breastfeeding rates by racial/ethnic groups varied, ranging from 6.6 percentage points in Vermont to 37.6 percentage points in North Dakota, as did the specific racial/ethnic groups with the highest and lowest rates. These state/territory-specific data highlight the variation that exists in breastfeeding disparities across the United States and can help public health practitioners and health departments identify groups on which to focus efforts. Targeting breastfeeding promotion programs on populations with lower breastfeeding rates might help reduce racial/ethnic disparities in breastfeeding initiation and improve infant nutrition and health.

Birth data from NVSS are a census of all live births in the United States collected by using the U.S. Standard Certificate of Live Birth.^§^ Birth certificate data include an infant nutrition item, determined from medical records, that indicates whether an infant received any breast milk or colostrum during the period between delivery and hospital discharge (*3*).^¶^ Data also include self-reported maternal race/ethnicity.**^,^^†† ^Analysis was restricted to data on infants who were alive when the birth certificate was completed and who were not transferred to another facility within 24 hours of delivery. Births during 2019 in 48 states, DC, Northern Mariana Islands, Guam, and Puerto Rico were included; breastfeeding data were not available for births in California, Michigan, American Samoa, or the U.S. Virgin Islands. Births in 48 states and DC (hereafter referred to as a state), representing 85.2% of U.S. live births, contributed to nearly national estimates (hereafter referred to as national). Infants for whom maternal race/ethnicity data were missing (n = 16,827, 0.5%) were included in national, state, and territorial estimates but excluded from estimates stratified by race/ethnicity.

The percentage of infants for whom breastfeeding was initiated was calculated overall and by maternal race/ethnicity at the national, state, and territorial levels. Assessed racial/ethnic groups included infants born to Hispanic, White, Black, Asian, American Indian or Alaska Native (AI/AN), Native Hawaiian/Other Pacific Islander (NH/OPI), and multiracial mothers. Racial/ethnic disparities were calculated in each state/territory as the percentage point difference between breastfeeding initiation among the racial/ethnic group with the highest initiation prevalence and each of the other groups. Because birth data are a census, no statistical tests were conducted. Data were suppressed for any racial/ethnic group with a denominator <50. Estimates for Guam, Northern Mariana Islands, and Puerto Rico were not considered in comparisons because each territory was excluded from national estimates and had data suppressed for three or more racial/ethnic groups. SAS (version 9.4; SAS Institute) was used for all data analyses.

The prevalence of initiation of breastfeeding for newborn infants was 84.1%, ranging from 94.8% in Oregon to 64.7% in Mississippi. Initiation rates varied by maternal race/ethnicity, ranging from 90.3% among infants of Asian mothers to 73.6% among infants of Black mothers. In 26 states (including DC), the breastfeeding initiation rate was lowest among infants of Black mothers; in 13 states, the rate was lowest among infants of AI/AN mothers (including in Maine, where rates were equally low among infants of multiracial mothers). Prevalence of breastfeeding initiation was highest among infants of Asian mothers in 36 states (including Vermont, where rates were equally high among infants of Black mothers) and highest among infants of White mothers in 10 states (including DC) (Table).

**TABLE:** Percentage of live infants not transferred to another facility for whom breastfeeding was initiated,* by state/territory and maternal race/ethnicity^†^ — National Vital Statistics System, 48 states,^§^ District of Columbia, Guam, Northern Mariana Islands, and Puerto Rico, 2019

Jurisdiction	No. of infants^¶^ (% initiating breastfeeding)								Largest disparity**
	Overall	Hispanic	White	Black	Asian	AI/AN	NH/OPI	Multiracial	
**United States^††^**	**3,129,646 (84.1)**	**665,584 (87.4)**	**1,686,505 (85.5)**	**492,852 (73.6)^§§^**	**164,602 (90.3)^¶¶^**	**25,807 (76.6)**	**7,843 (80.2)**	**69,626 (83.1)**	**16.7**
Alabama	56,054 (69.6)	4,730 (64.2)	32,031 (77.2)	17,285 (56.1)^§§^	863 (84.6)^¶¶^	140 (73.6)	—***	971 (71.5)	28.5
Alaska	9,492 (92.9)	761 (95.9)	4,685 (96.3)^¶¶^	288 (94.4)	569 (81.0)	1,812 (88.5)	293 (76.1)^§§^	919 (94.7)	20.2
Arizona	78,613 (88.9)	33,426 (87.9)	31,629 (91.1)	4,489 (84.5)	2,846 (93.3)^¶¶^	3,739 (84.7)	218 (80.7)^§§^	1,842 (86.6)	12.6
Arkansas	34,123 (73.7)	3,769 (81.9)	21,994 (78.0)	6,052 (52.3)^§§^	698 (85.0)^¶¶^	218 (72.9)	506 (67.0)	654 (74.3)	32.7
Colorado	62,552 (92.8)	18,032 (90.4)	35,906 (94.6)^¶¶^	3,009 (88.4)	2,552 (93.3)	375 (85.1)^§§^	167 (91.0)	1,532 (92.3)	9.5
Connecticut	34,558 (87.4)	8,861 (85.5)	18,616 (88.4)	4,096 (84.0)^§§^	2,253 (93.1)^¶¶^	—	—	687 (84.6)	9.1
Delaware	10,717 (80.5)	1,723 (83.8)	5,332 (82.2)	2,687 (72.7)	608 (93.8)^¶¶^	—	—	329 (72.6)^§§^	21.2
DC	13,092 (84.2)	1,846 (82.5)	4,799 (97.1)^¶¶^	5,313 (71.2)^§§^	808 (95.5)	—	—	300 (88.7)	25.9
Florida	215,942 (87.2)	67,193 (91.1)	91,783 (87.7)	46,750 (79.6)^§§^	6,944 (92.9)^¶¶^	224 (81.7)	161 (88.8)	2,839 (86.6)	13.3
Georgia	124,711 (83.1)	18,259 (90.1)	53,889 (84.5)	43,241 (77.0)^§§^	5,777 (93.9)^¶¶^	93 (83.9)	99 (81.8)	2,472 (84.6)	16.9
Hawaii	16,583 (89.6)	2,567 (92.1)	3,182 (96.8)^¶¶^	422 (96.2)	4,287 (86.3)	—	1,671 (78.2)^§§^	4,387 (90.3)	18.6
Idaho	21,463 (92.2)	3,653 (90.1)	16,448 (92.8)	258 (94.6)	347 (93.7)	224 (78.6)^§§^	74 (97.3)^¶¶^	401 (91.8)	18.7
Illinois	134,795 (83.1)	29,555 (86.4)	70,731 (85.2)	22,406 (67.7)^§§^	9,091 (93.1)^¶¶^	74 (81.1)	—	1,904 (81.5)	25.4
Indiana	80,077 (82.9)	8,316 (85.4)^¶¶^	57,777 (84.1)	10,077 (74.1)^§§^	2,250 (84.0)	58 (75.9)	59 (79.7)	1,516 (81.1)	11.3
Iowa	36,876 (83.1)	3,821 (81.0)	28,030 (84.9)	2,861 (69.6)^§§^	1,102 (85.5)^¶¶^	259 (73.7)	156 (73.7)	645 (78.3)	15.9
Kansas	36,442 (89.6)	6,290 (87.1)	25,216 (90.9)	2,626 (84.3)	1,250 (93.4)^¶¶^	131 (84.7)	66 (77.3)^§§^	797 (85.3)	16.1
Kentucky	49,321 (71.7)	3,216 (82.1)	39,288 (71.1)	4,650 (67.0)^§§^	1,007 (86.8)^¶¶^	—	58 (79.3)	924 (69.9)	19.8
Louisiana	56,966 (71.0)	4,851 (82.2)	28,945 (78.9)	21,027 (56.5)^§§^	1,136 (87.0)^¶¶^	239 (70.7)	—	705 (74.3)	30.5
Maine	11,148 (89.4)	237 (85.2)	9,844 (89.4)	514 (92.0)	211 (93.4)^¶¶^	87 (83.9)^§§^	—	249 (83.9)^§§^	9.5
Maryland	66,056 (87.1)	12,166 (94.1)	27,898 (86.4)	19,537 (82.0)^§§^	4,547 (95.4)^¶¶^	67 (83.6)	—	1,582 (84.5)	13.4
Massachusetts	68,897 (88.4)	14,027 (86.9)	39,346 (88.3)	6,776 (90.5)	6,197 (91.4)^¶¶^	78 (84.6)^§§^	—	1,491 (85.5)	6.8
Minnesota	62,276 (89.7)	4,867 (90.2)	42,110 (91.9)^¶¶^	7,690 (87.5)	5,032 (78.9)	858 (67.5)^§§^	52 (86.5)	1,545 (85.8)	24.4
Mississippi	35,022 (64.7)	1,620 (71.7)	17,195 (74.5)	15,270 (52.5)	437 (83.3)^¶¶^	219 (49.3)^§§^	—	255 (70.6)	34.0
Missouri	69,799 (79.7)	4,228 (81.0)	50,967 (82.2)	10,019 (65.5)^§§^	1,731 (88.9)^¶¶^	150 (72.0)	219 (74.0)	2,224 (78.6)	23.4
Montana	10,929 (90.6)	611 (90.0)	8,737 (93.1)^¶¶^	61 (86.9)	116 (89.7)	1,050 (70.2)^§§^	—	324 (88.6)	22.9
Nebraska	24,724 (88.6)	4,145 (85.5)	17,316 (90.5)	1,570 (78.6)	835 (91.1)^¶¶^	218 (74.3)^§§^	—	617 (84.1)	16.8
Nevada	33,410 (80.0)	12,610 (81.9)	11,895 (84.2)^¶¶^	4,263 (64.1)^§§^	2,542 (81.2)	241 (83.4)	363 (72.2)	1,386 (77.4)	20.1
New Hampshire	11,609 (90.8)	728 (90.1)	9,979 (90.6)	253 (94.9)	454 (96.5)^¶¶^	—	—	129 (85.3)^§§^	11.2
New Jersey	95,969 (79.7)	26,746 (80.9)	43,923 (81.0)	12,877 (70.9)^§§^	10,317 (83.2)^¶¶^	64 (73.4)	—	1,075 (78.4)	12.3
New Mexico	21,040 (86.1)	11,503 (84.8)	5,728 (88.9)	332 (81.6)^§§^	374 (91.2)^¶¶^	2,688 (86.1)	—	370 (84.9)	9.6
New York	219,529 (87.9)	49,898 (90.4)^¶¶^	107,699 (87.4)	31,926 (84.4)	24,683 (89.8)	358 (82.1)	71 (81.7)^§§^	3,195 (82.3)	8.7
North Carolina	119,198 (81.6)	19,084 (88.1)	62,586 (84.6)	27,785 (70.8)	4,957 (89.0)^¶¶^	1,429 (52.1)^§§^	127 (82.7)	3,130 (79.4)	36.9
North Dakota	11,702 (85.0)	733 (83.8)	8,499 (88.2)	756 (86.0)	251 (91.6)^¶¶^	847 (54.0)^§§^	—	358 (81.3)	37.6
Ohio	128,555 (76.0)	7,428 (77.8)	91,498 (77.2)	21,415 (68.8)^§§^	4,237 (87.1)^¶¶^	98 (78.6)	101 (72.3)	3,600 (70.9)	18.3
Oklahoma	46,523 (81.8)	7,284 (81.8)	25,823 (84.7)	3,961 (71.6)	1,215 (85.8)^¶¶^	4,485 (74.8)	218 (62.4)^§§^	3,478 (80.2)	23.4
Oregon	41,473 (94.8)	8,019 (94.7)	27,456 (94.9)	1,006 (94.9)	2,374 (97.1)^¶¶^	398 (88.4)^§§^	336 (91.1)	1,599 (93.7)	8.7
Pennsylvania	128,439 (82.2)	16,017 (81.8)	84,758 (83.0)	16,922 (76.9)^§§^	6,002 (91.1)^¶¶^	77 (79.2)	—	3,073 (77.7)	14.2
Rhode Island	10,592 (67.7)	2,919 (56.3)	5,959 (75.2)	828 (54.5)^§§^	528 (76.1)^¶¶^	—	—	272 (56.6)	21.6
South Carolina	52,493 (78.1)	5,414 (86.5)	28,919 (82.8)	15,609 (66.0)^§§^	1,001 (90.6)^¶¶^	100 (68.0)	53 (73.6)	1,275 (77.1)	24.6
South Dakota	11,966 (80.7)	679 (77.5)	8,758 (84.7)^¶¶^	421 (79.1)	208 (72.6)	1,479 (61.2)^§§^	—	398 (76.1)	23.5
Tennessee	84,201 (81.1)	8,596 (85.3)	55,082 (83.0)	16,540 (71.3)^§§^	1,846 (92.0)^¶¶^	97 (87.6)	73 (87.7)	1,588 (83.2)	20.7
Texas	376,721 (88.5)	179,268 (88.4)	124,558 (90.4)	47,113 (81.5)^§§^	19,806 (95.2)^¶¶^	693 (87.6)	572 (86.5)	4,349 (87.8)	13.7
Utah	47,200 (86.2)	8,194 (81.8)	33,650 (88.8)^¶¶^	608 (73.4)	1,088 (85.1)	354 (75.7)	447 (69.1)^§§^	1,073 (88.6)	19.7
Vermont	5,062 (91.3)	124 (93.5)	4,555 (91.0)^§§^	126 (97.6)^¶¶^	123 (97.6)^¶¶^	—	—	82 (93.9)	6.6
Virginia	95,415 (86.2)	14,294 (92.1)	51,270 (87.3)	20,448 (76.3)^§§^	7,351 (94.5)^¶¶^	142 (88.0)	124 (89.5)	1,706 (87.7)	18.2
Washington	82,930 (94.6)	15,885 (92.8)	46,246 (95.3)	3,689 (94.4)	8,665 (96.7)^¶¶^	996 (88.8)^§§^	1,177 (91.0)	3,557 (94.7)	7.9
West Virginia	18,187 (64.8)	359 (77.4)	16,590 (64.6)	591 (59.4)^§§^	170 (86.5)^¶¶^	—	—	387 (64.6)	27.1
Wisconsin	60,439 (81.1)	6,270 (79.0)	42,876 (86.6)^¶¶^	6,357 (53.8)^§§^	2,851 (68.4)	581 (69.2)	—	1,329 (76.1)	32.8
Wyoming	5,765 (83.9)	762 (77.3)	4,504 (85.6)	52 (65.4)^§§^	65 (87.7)^¶¶^	170 (70.6)	—	106 (83.0)	22.3
NMI	669 (97.3)	—	—	—	252 (97.6)^¶¶^	—	377 (97.3)^§§^	—	0.3
Guam	2,661 (80.6)	—	175 (90.9)^¶¶^	—	721 (78.5)^§§^	—	1,607 (79.9)	86 (84.9)	12.4
Puerto Rico	19,910 (93.6)	19,432 (93.6)^¶¶^	401 (92.3)^§§^	—	—	—	—	—	1.3

**Abbreviations:** AI/AN = American Indian/Alaska Native; DC = District of Columbia; NH/OPI = Native Hawaiian/Other Pacific Islander; NMI = Northern Mariana Islands.

* Excludes infants transferred to another facility within 24 hours of delivery and those who died before completion of the birth certificate.

^†^ All racial/ethnic groups are non-Hispanic unless otherwise noted.

^§^ Includes all states except California and Michigan.

^¶^ Denominators might not sum to total because of missing maternal race/ethnicity data.

** Largest disparity is defined as the percentage point difference in breastfeeding initiation prevalence between the racial/ethnic groups with the highest and lowest initiation prevalence.

^††^ United States estimates include data from 48 states and DC but exclude data from territories.

^§§^ Racial/ethnic group with the lowest breastfeeding initiation prevalence.

^¶¶^ Racial/ethnic group with highest breastfeeding initiation prevalence.

*** Data were suppressed for all racial/ethnic groups with denominators <50.

Generally, racial/ethnic disparities in breastfeeding initiation were larger in states with lower overall breastfeeding initiation rates (Figure 1). Nationally, the largest racial/ethnic disparity in breastfeeding initiation was 16.7 percentage points (higher for infants of Asian mothers than for infants of Black mothers) and ranged from 6.6 percentage points in Vermont (higher for infants of Black and Asian mothers than for infants of White mothers) to 37.6 percentage points in North Dakota (higher for infants of Asian mothers than for infants of AI/AN mothers). The largest disparity exceeded 20 percentage points in 22 states (including DC) and exceeded 30 percentage points in six states (Table) (Figure 2). The racial/ethnic groups that corresponded to the largest disparity varied across states. The largest disparities were most commonly observed between infants of Asian mothers and infants of Black mothers (in 22 states) followed by infants of Asian mothers and infants of AI/AN mothers (in eight states) (Table).

**FIGURE 1:**
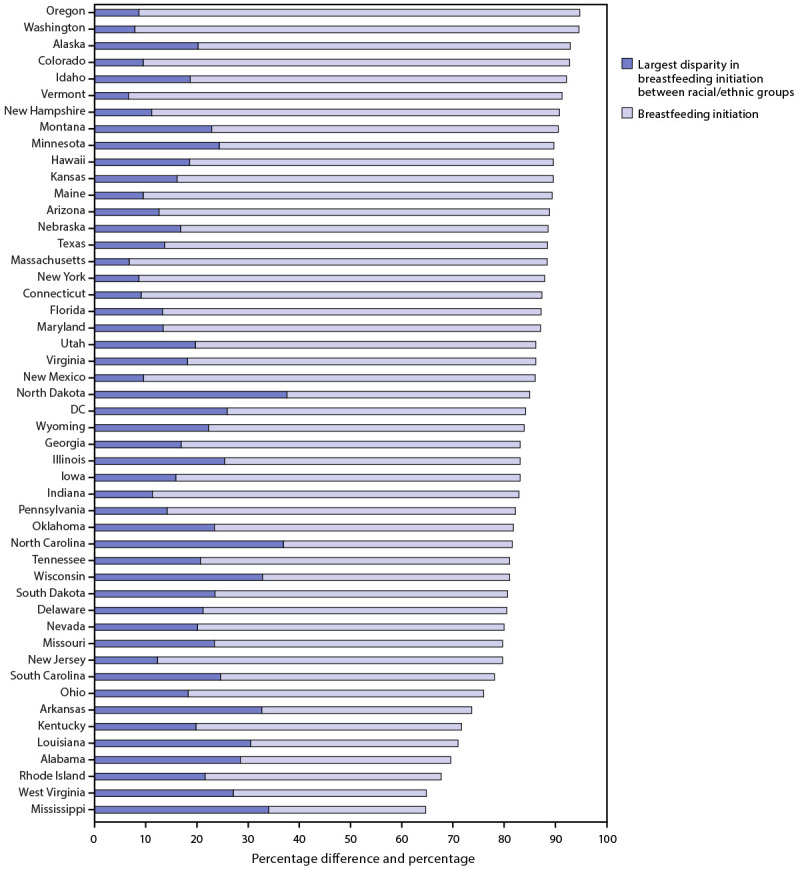
Breastfeeding initiation and largest disparity in breastfeeding initiation between racial/ethnic groups,* by state^†^ — National Vital Statistics System, 48 states and the District of Columbia, 2019 **Abbreviation**: DC = District of Columbia. *Breastfeeding initiation is measured as a percentage. Largest disparity in breastfeeding initiation between racial/ethnic groups is measured as a percentage difference. ^†^ Includes all states except California and Michigan. California does not report breastfeeding initiation data to the National Vital Statistics System. Michigan uses nonstandard wording for the breastfeeding initiation item on the birth certificate, which prevents comparison of data to other states.

**FIGURE 2:**
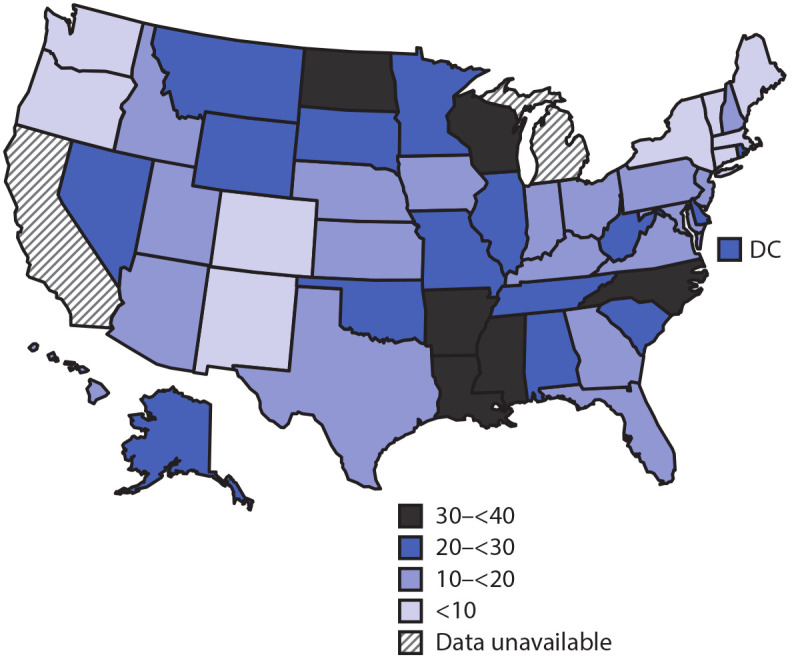
Largest disparity in breastfeeding initiation between racial/ethnic groups, by percentage point difference ─ National Vital Statistics System, 48 states and the District of Columbia, 2019 **Abbreviation**: DC = District of Columbia.

## Discussion

Although most mothers in the United States initiated breastfeeding, approximately one in six infants born in 2019 did not receive any breast milk, and disparities in initiation existed. Initiation rates varied geographically, with large racial/ethnic disparities existing both nationally and at state and territorial levels. Furthermore, states with lower breastfeeding initiation rates generally had a higher prevalence of racial/ethnic breastfeeding disparities than did states with higher initiation rates. Although breastfeeding initiation rates were generally higher among infants of Asian and White mothers and lower among infants of AI/AN and Black mothers, this was not true in all states. Likewise, the magnitude of disparities in breastfeeding initiation between the highest and lowest initiating racial/ethnic groups varied considerably from state to state. These data show that disparities are often state-specific and suggest that efforts tailored to address each state’s specific disparities might be needed.

Breastfeeding is associated with reduced risk for various infections, necrotizing enterocolitis, sudden infant death syndrome, type 1 diabetes, and obesity among infants, and with reduced risk for high blood pressure, type 2 diabetes, ovarian cancer, and breast cancer among mothers.^§§^ Because Black and AI/AN populations are at increased risk for many of these health outcomes,^¶¶^^,^***^,^^†††^^,^^§§§^^,^^¶¶¶^ lower rates of breastfeeding initiation among these groups are particularly concerning. Racial/ethnic disparities in meeting breastfeeding duration and exclusivity recommendations (*1*) can contribute to high disease prevalence and increased associated costs. For example, a recent study estimated that 1.3 times the number of excess cases of maternal hypertension among Black mothers compared with White mothers and 3.3 times the number of excess cases of necrotizing enterocolitis among Black infants compared with White infants can be attributed to lower rates of breastfeeding exclusivity and duration (*4*). Although this report includes data only on breastfeeding initiation, disparities in breastfeeding duration and exclusivity result, in part, from differences in breastfeeding initiation (*5*).

Efforts are needed to increase overall breastfeeding initiation and reduce racial/ethnic disparities at the national, state, and territorial levels. Hospitals can implement evidence-based maternity care policies and practices that support breastfeeding. Research has found that implementation of programs such as the Ten Steps to Successful Breastfeeding improves overall breastfeeding outcomes and decreases racial/ethnic inequities (*6*,*7*). Further, state and territorial health departments could consider developing culturally relevant initiatives or refocusing current breastfeeding promotion efforts to better target their populations at highest risk. CDC currently funds efforts in 16 states**** to implement evidence-based strategies to improve nutrition and physical activity, including breastfeeding.^††††^ In partnership with the Association of State and Territorial Health Officials, CDC is supporting nine of these states^§§§§^ in efforts to develop and implement innovative strategies to promote equity and reduce disparities in breastfeeding (*8*).

CDC uses the National Immunization Survey (NIS) for routine surveillance of breastfeeding initiation, duration, and exclusivity.^¶¶¶¶^ However, relatively small sample sizes prohibit routine estimation of breastfeeding by race/ethnicity at the state and territorial levels. NVSS has data only on breastfeeding initiation, but as a census of all births, it has a robust sample size, which allows examination of breastfeeding disparities at the state and territorial levels. National breastfeeding initiation rates calculated from 2019 birth certificate data are comparable to rates estimated from NIS survey data (84.1% among infants born in 2017). Initiation rates are also generally similar across both data sources for most states and racial/ethnic groups.*****

The findings in this report are subject to at least four limitations. First, birth certificates do not include information on exclusivity and duration of breastfeeding, which are important indicators of optimal infant nutrition. Second, breastfeeding initiation data might be misclassified. Although a comparison of birth certificates to medical records across eight hospitals in two states found high sensitivity for breastfeeding initiation (90.7% and 96.2%), moderate false discovery rates (19% and 16%) suggest that discrepancies might exist between medical records and birth certificates (*9*); however, overall rates are generally consistent with other national data sources. Further, no true gold standard exists for comparison to birth certificate data, and data from which previous comparisons have been made are limited and nearly a decade old. Third, birth certificate data reliability and validity are not known to have been assessed across racial/ethnic groups. Misclassification of breastfeeding data might vary by race/ethnicity. Finally, estimates are not nationally representative because births from California and Michigan (representing 14.8% of U.S. births) were not included in analyses.

Although breastfeeding can help reduce risks for several maternal and infant health conditions, infants from some racial/ethnic minorities who are already at the highest risk for these conditions are often among the least likely to be breastfed. These data might be useful to state and territorial public health practitioners in identifying specific racial/ethnic disparities on which to focus efforts to improve breastfeeding support. Implementation of evidence-based maternity care policies and practices supportive of breastfeeding and targeted breastfeeding programs focusing on populations at highest risk for low breastfeeding initiation might help reduce racial/ethnic disparities in breastfeeding initiation, improve infant nutrition, and reduce maternal and infant.

**Summary**

**What is already known about this topic?**

Although rates of breastfeeding initiation have increased during the past decade, racial/ethnic disparities in breastfeeding persist.

**What is added by this report?**

Birth certificate data indicate that the magnitude of racial/ethnic disparities in breastfeeding initiation varies across states as do the racial/ethnic groups corresponding to each state’s largest disparity.

**What are the implications for public health practice?**

Efforts are needed to increase breastfeeding initiation and reduce racial/ethnic disparities. Because disparities are state-specific, efforts tailored to address each state’s disparities might be needed. Maternity care policies and practices supportive of breastfeeding and breastfeeding programs that target highest risk populations might help increase initiation, reduce disparities, and improve infant nutrition.

## Footnotes

* All racial/ethnic groups are non-Hispanic unless otherwise noted.

† California does not report breastfeeding initiation data to NVSS. Michigan uses nonstandard wording for the breastfeeding initiation item on the birth certificate,which prevents comparison of data to other states.

§
https://www.cdc.gov/nchs/data/dvs/birth11-03final-ACC.pdf

¶ Analyses were not limited to births occurring in hospitals. NVSS provides birth certificate completion instructions to facilities, but breastfeeding information is also collected for births occurring in other locations (e.g., freestanding birth centers, clinics, doctors’ offices, and homes) via the birth certificate.

**
https://www.cdc.gov/nchs/nvss/revisions-of-the-us-standard-certificates-and-reports.htm?CDC_AA_refVal=https%3A%2F%2Fwww.cdc.gov%2Fnchs%2Fnvss%2Fvital_certificate_revisions.htm

††
https://www.cdc.gov/nchs/data/dvs/moms-worksheet-2016-508.pdf

§§
https://www.cdc.gov/breastfeeding/about-breastfeeding/why-it-matters.html

¶¶
https://www.cdc.gov/sids/data.htm

***
https://www.cdc.gov/obesity/data/childhood.html

†††
https://www.cdc.gov/bloodpressure/facts.htm

§§§
https://www.cdc.gov/diabetes/basics/quick-facts.html

¶¶¶
https://gis.cdc.gov/Cancer/USCS/DataViz.html

**** Alaska, Arkansas, California, Colorado, Connecticut, Illinois, Kentucky, Minnesota, Missouri, New York, North Carolina, Ohio, Pennsylvania, Texas, Utah, and Washington.

††††
https://www.cdc.gov/nccdphp/dnpao/state-local-programs/span-1807/index.html

§§§§ Alaska, Arkansas, Colorado, Illinois, Missouri, Ohio, Pennsylvania, Utah, and Washington.

¶¶¶¶
https://www.cdc.gov/breastfeeding/data/nis_data/

*****
https://www.cdc.gov/breastfeeding/data/nis_data/results.html
