# *Erratum:* Vol. 70, No. 21

**DOI:** 10.15585/mmwr.mm7034a5

**Published:** 2021-08-27

In the report “Racial and Ethnic Disparities in Breastfeeding Initiation ― United States, 2019,” on page 774, the last sentence should have read, “Implementation of evidence-based maternity care policies and practices supportive of breastfeeding and targeted breastfeeding programs focusing on populations at highest risk for low breastfeeding initiation might help reduce racial/ethnic disparities in breastfeeding initiation, improve infant nutrition, and reduce maternal and infant **morbidity**.”

